# Diagnosis and treatment strategies for cardiogenic shock in Takotsubo syndrome with a temporary left ventricular assist device—A single-center study

**DOI:** 10.1016/j.jhlto.2026.100520

**Published:** 2026-02-16

**Authors:** Anna Huang, Johanna K.R. von Mackensen, Vanessa I.T. Zwaans, Carla L. Schuering, Jasper Iske, Leonard Pitts, Julius Kaemmel, Gaik Nersesian, Cheng-Ying Chiu, Christoph T. Starck, Joerg Kempfert, Stephan Jacobs, Volkmar Falk, Evgenij V. Potapov, Leonhard Wert

**Affiliations:** a Department of Cardiothoracic and Vascular Surgery, Deutsches Herzzentrum der Charité—Medical Heart Center of Charité and German Heart Institute, Berlin, Germany; b Charité, Universitätsmedizin Berlin, Corporate Member of Freie Universität Berlin, Humboldt-Universität zu Berlin, and Berlin Institute of Health, Berlin, Germany; c Department of Cardiology, Angiology and Intensive Care Medicine, Medical Heart Center of Charité and German Heart Institute, Berlin, Germany; d DZHK (German Center for Cardiovascular Research), Partner Site, Berlin, Germany; e Department of Health Sciences and Technology, ETH Zürich, Zurich, Switzerland

**Keywords:** Takotsubo syndrome, stress cardiomyopathy, mechanical circulatory support, Impella, ECLS, ECMO

## Abstract

**Background:**

Takotsubo syndrome (TTS) is a reversible form of acute heart failure characterized by apical ballooning, which may lead to cardiogenic shock (CS). When CS occurs, mechanical circulatory support (MCS) may be used to bridge patients until cardiac recovery. However, the role, optimal timing, and choice of MCS remain unclear.

**Methods:**

On February 22, 2024, a search of the clinic’s electronic database was performed for patients diagnosed with TTS. The search yielded 47 patients, with 9 patients receiving MCS. Individual patient data concerning these 9 cases were extracted and analyzed.

**Results:**

Of 9 TTS patients with MCS, 5 were supported by an Impella system, with the remaining 4 patients receiving a combination of Impella and extracorporeal membrane oxygenation. Left ventricular ejection fraction measured at admission ranged from 10% to 36%, with 4 patients showing full recovery of left ventricular ejection function. Three patients died in the hospital and 1 patient remains on long-term support with a HeartMate 3 left ventricular assist device.

**Conclusions:**

This retrospective single–center study on the use of MCS in TTS management supports results from previous studies reporting on the benefit of Impella therapy. Our experience shows that Impella support provides stabilization of CS and unloading of the left ventricle, allowing full recovery of left ventricular function and reversing TTS findings. For patients who do not adequately respond to Impella support alone, escalation to a combination of Impella and extracorporeal membrane oxygenation strategy is feasible to further improve cardiac output, while protecting the left ventricle from the increased afterload**.**

## Background

Takotsubo syndrome (TTS) or “stress cardiomyopathy” is a reversible form of acute heart failure and is characterized by apical ballooning of the left ventricle with compensatory basal hypercontractility resulting in left ventricular (LV) dysfunction.^1^ TTS commonly presents with symptoms of acute coronary syndrome (ACS) and affects mostly postmenopausal women.^2^, ^3^ The underlying pathophysiology is complex and not well understood; however, cardiac stunning induced by excessive catecholamine levels seems to play a central role.^2^, ^4^ Due to the overlap in clinical presentation with ACS and an incomplete knowledge of diagnostic criteria, such as the possibility of ACS and TTS as coexisting conditions, TTS remains an under-recognized diagnosis.^5^

Instruments used in the process of diagnosing TTS are the Mayo Clinic Diagnostic Criteria^6^ and the InterTAK Diagnostic Criteria.^7^ These criteria require exclusion of significant coronary artery disease (CAD), echocardiographic findings of hypo-, a-, or dyskinesia extending beyond a single coronary vascular territory, as well as consideration of typical emotional or physical triggers.^6^, ^7^

When cardiac output is significantly reduced, TTS can lead to cardiogenic shock (CS), with incidences of 4% to 20% and reported in-hospital mortality rates of 17% to 30%.^2^, ^8^, ^9^ In the setting of TTS-CS, mechanical circulatory support (MCS) may be used as a bridge-to-recovery strategy. The implantation of a temporary microaxial LV assist device (Impella, Abiomed, Danvers, MA) in patients with CS is an established technique.^10^ However, literature on the use of such MCS devices in the context of TTS-CS is scarce and largely comprises case reports, case series, and a few systematic review articles.^10^, ^11^, ^12^, ^13^, ^14^, ^15^ The role, timing, and choice of MCS are subject to ongoing discussion, particularly given the catecholamine sensitivity of TTS and the benefit of ventricular unloading devices in minimizing the use of inotropes.^2^, ^4^, ^16^, ^17^, ^18^

We evaluated all patients who were diagnosed with TTS at our clinic and received MCS with regard to clinical characteristics, management strategies, and outcomes.

## Patients and methods

### Study design

This is a single-center, retrospective study for which data were collected from 9 patients diagnosed with TTS who received MCS. The institutional review board or equivalent ethics committee of the authors’ institutions approved the study protocol and publication of data. The study was approved by the institutional review board at Charité – Universitätsmedizin Berlin (approval number: EA1/196/21). The patients provided informed written consent for the publication of the study data.

### Patient inclusion criteria

An electronic search of the clinic’s patient database was performed on February 22, 2024, with the following search words: “takotsubo,” “tako-tsubo,” “stress cardiomyopathy” (in German), “apical ballooning.” The resulting patient list was then screened for inclusion criteria.

Patient inclusion criteria were:

1. TTS diagnosis according to the InterTAK Diagnostic Criteria^7^
2. Support with MCS: extracorporeal membrane oxygenation (ECMO), Impella, and intra-aortic balloon pump.

### Data extraction

Individual patient data were extracted from the electronic patient database by the second author and checked for accuracy by the last author. The extracted data included patient birthdate and sex, underlying health conditions, MCS strategy, vital signs before circulatory support (systolic/diastolic blood pressure or mean arterial pressure, heart rate), respiratory failure, use of inotropes before MCS, prior angiography, cardiac arrest before MCS, new electrocardiogram (ECG) abnormalities (ST elevation, ST depression, and T-wave inversion), new arrhythmias, left ventricular ejection fraction (LVEF) (before MCS, on MCS and in follow-up), baseline lactate, time to weaning of inotropes under MCS, time on MCS and follow-up (last documented date after implantation, LV recovery, and survival).

### Statistical analysis

Available data were extracted from the eligible patients’ histories and included in the statistical analysis. Categorical variables are presented as N (%), and continuous variables are summarized as mean ± standard deviation (SD). Descriptive statistical analysis was performed using the data analysis tool R, Version 4.02 (R Core Team, Vienna, Austria).

## Results

Forty-seven patients whose differential diagnoses included TTS were identified in the electronic patient database of this clinic. Nine patients received MCS. Of these, 5 patients were supported by an Impella system. Another 4 patients received a combination of Impella and ECMO (ECMELLA) support. Patient baseline data are summarized in Table 1 with the detailed MCS strategy described further below in Table 2.

**Table 1:** Demographic and Baseline Characteristics of Patients Who Received MCS for TTS at Our Clinic

Variable	Value
Female, N (%)	9 (100.0)
Age at TTS diagnosis, mean±SD	60.4±15.5
BMI (kg/m^2^), mean±SD	23.9±2.7
LVEF at admission (%), mean±SD	28.2±19.4
Lactate at admission (mg/dl), mean±SD	31.9±37.3
Cardiovascular risk factors, N (%)	
Type II diabetes mellitus	1 (11.1)
Arterial hypertension	4 (44.4)
Dyslipidemia	4 (44.4)
Nicotine abuse	3 (33.3)
Pre-existing conditions, N (%)	
Coronary artery disease	4 (44.4)
Peripheral arterial disease	3 (33.3)
Kidney disease	4 (44.4)
Chronic heart failure, N (%)	
NYHA I	2 (22.2)
NYHA IV	2 (22.2)
EuroSCORE II (%), mean±SD	25.4±19.2
Rethoracotomy, N (%)	1 (11.1)
Neurological complication, N (%)	4 (44.4)
Survival, N (%)	6 (66.7)
Follow-up time (days), mean±SD	23 (74.2)
MCS strategy, N (%)	
Impella	5 (55.6)
ECMELLA	4 (44.4)

Abbreviations: BMI, body mass index; ECMELLA, combination of left ventricular unloading by Impella devices and extracorporeal life support; LVEF, left ventricular ejection fraction; MCS, mechanical circulatory support; NYHA, New York Heart Association; SD, standard deviation; TTS, Takotsubo syndrome.

## Case 1

A 67-year-old female patient with multiple vascular diseases (peripheral artery disease, arterial hypertension, and chronic kidney disease) presented with angina pectoris. She was initially diagnosed with CAD and treated with stent implantation. However, additional wall motion abnormalities were observed in areas unrelated to the stenosed vessels, along with apical ballooning. MCS with an Impella 2.5 was initiated for 4 days, increasing cardiac output from 2.5 to 4.7 liters/min and leading to stabilization of LVEF.

## Case 2

**Figure 1:**
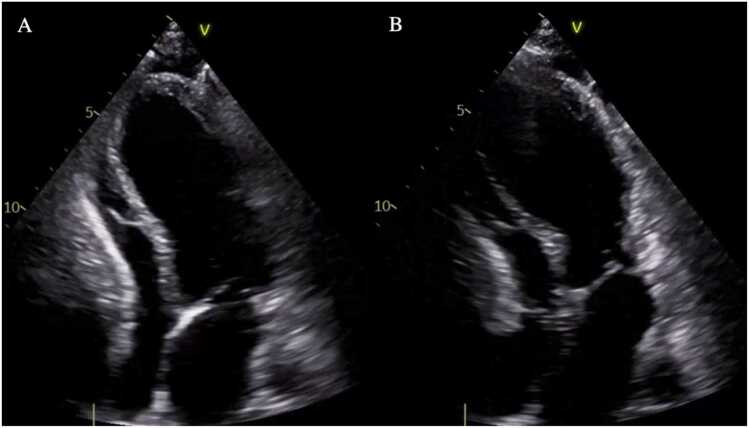
Transthoracic echocardiogram, 2-chamber view of patient 2 showing typical Takotsubo findings. (A) End-diastolic image. (B) End-systolic image showing akinesia of the mid and apical segments.

**Figure 2:**
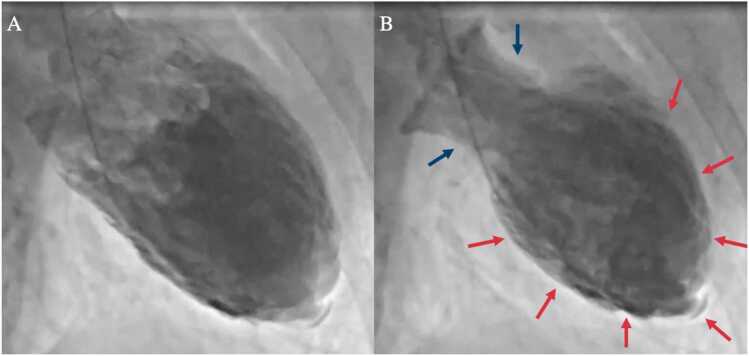
Left ventriculography of patient 2 showing typical Takotsubo findings. (A) Disatolic phase. (B) Systolic phase with image showing basal hyperkinesia (blue arrows) and akinesia of the mid and apical segments (red arrows).

A 53-year-old woman with no significant medical history, but with psychological stressors, presented to an external hospital with presyncope and vertigo. CAD was ruled out. The patient was transferred to our hospital with TTE showing an LVEF of 25% and apical dyskinesia (Figures 1 and 2; Videos 1-10). An Impella 5.5 system was implanted via the right axillary artery for temporary MCS, establishing a blood flow of 4 liters/min which led to rapid hemodynamic stabilization. The patient was extubated on the first postoperative day, and Impella support was maintained for 4 days and 11 hours. After Impella explantation, TTE showed an LVEF of 45%, minimal mitral and tricuspid regurgitation, and remaining apical akinesia. Heart failure therapy was initiated with sacubitril/valsartan and the patient was discharged to a rehabilitation facility 9 days after the initial presentation.

## Case 3

A 77-year-old female patient had previously undergone minimally invasive surgical mitral valve replacement. After an unremarkable course, the patient developed acute heart failure 11 days postsurgery with lung edema and pleural effusion. TTE revealed an LVEF of 33% and showed the typical morphology of newly developed TTS. An Impella 5.5 system implanted via a transaxillary approach without complications led to immediate stabilization of the hemodynamic situation. Over the postoperative course, the patient developed a bronchopulmonary infection with clinical improvement under antibiotic therapy. Due to persistent atrial flutter, electrical cardioversion was attempted 3 days after Impella implantation, successfully restoring sinus rhythm. The patient experienced progressive pleural effusion, warranting chest tube insertion. Afterward, electrical cardioversion was performed again to convert the atrial flutter to sinus rhythm. With heart function improving, the patient was weaned and the Impella 5.5 was explanted after 5 days and 18 hours. A routine chest X-ray image revealed atelectasis formation on the right side, prompting escalation of conventional oxygen therapy to noninvasive ventilation. The patient was discharged home 35 days after the mitral valve replacement (22 days after TTS diagnosis).

## Case 4

A 57-year-old woman with known small–cell lung cancer under immunotherapy presented to the emergency room of an external hospital with fever, dysuria, and general fatigue. A urinary tract infection and influenza A pneumonia were confirmed, and the patient initially received pivmecillinam and oseltamivir. Due to suspected superinfection of the influenza-pneumonia, antibiotic therapy was escalated to ampicillin/sulbactam. Respiratory failure and shock prompted a transfer to the intensive care unit, where the patient was intubated, and antibiotic therapy was further escalated to piperacillin/tazobactam.

TTE revealed a severely limited LV function and apical akinesia with laboratory parameters indicative of non-ST-segment elevation myocardial infarction. CAD was ruled out via coronary angiogram, with levocardiography showing signs of TTS.

Over the next 4 days, the patient’s condition further deteriorated to the clinical picture of combined septic CS with increasing catecholamine demand and continued lactic acidosis. Subsequently, the patient was transferred to our clinic, where she underwent Impella 5.5 implantation via the right axillary artery. Due to the development of hemiplegia on the left side, cranial computed tomography (cCT) imaging was performed again, showing signs of a middle cerebral artery infarct on the right side on the second postoperative day. Over the postoperative course, antibiotic therapy was de-escalated and heart function normalized, enabling Impella explantation on the eighth postoperative day.

On the ninth postoperative day, dilatational tracheotomy was performed due to an unsuccessful spontaneous breathing trial. The patient received 2 cycles of tracheal high-flow therapy per day for 4 hours at 40 liters/min with 40% fraction of inspired oxygen (FiO_2_) and pressure support ventilation during the night.

The patient was discharged to a neurological early rehabilitation facility almost 1 month after the initial presentation.

## Case 5

A female patient aged 76 presented with palpitations, dyspnea, inner restlessness, and general discomfort. The ECG at admission revealed atrial fibrillation. Coronary angiography showed 2-vessel CAD without relevant stenosis. Dilated cardiomyopathy and TTS with severely impaired LV function were concomitantly diagnosed. One day later, acute kidney injury and catecholamine dependency necessitated Impella implantation via the left axillary artery.

On the first postoperative day, Impella explantation was performed. Transesophageal echocardiography (TEE) showed an LVEF of 55%, grade I mitral regurgitation, and grade II tricuspid regurgitation.

On the sixth postoperative day, the patient displayed anisocoria with the cCT showing intracranial hemorrhage without the possibility of neurosurgical intervention. The patient expired on the seventh postoperative day.

## Case 6

A 69-year-old woman presented to an external hospital with CS. CAD was ruled out via coronary angiography. Impaired systolic pump function with an LVEF of 35.5% and apical ballooning as well as basal hypokinesia resulted in the diagnosis of TTS. The same night, the patient suffered circulatory arrest with return of spontaneous circulation. Acute kidney injury necessitated the use of renal replacement therapy. The following day, extracorporeal life support (ECLS) was established since the patient showed no improvement in her hemodynamic condition and remained on high–dosage catecholamine therapy. After being transferred to our clinic, the patient underwent additional Impella implantation. Despite adequate organ perfusion, the patient displayed no awakening reaction. ECMELLA weaning was not possible, and the patient expired 11 days after the initial presentation in our department.

## Case 7

A 34-year-old pregnant woman underwent emergency surgery for type A aortic dissection with implantation of an ascending aorta prosthesis and David procedure, accompanied by a simultaneous cesarean section. After initial postoperative stabilization, cardiac function increasingly deteriorated over the further course. TEE revealed typical signs of TTS with apical ballooning and an LVEF of 17%. Emergency Impella implantation was then performed via the right axillary artery, initially resulting in an improved LVEF with blood flow of up to 4 liters/min. Ten days later, pump thrombosis with low cardiac output requiring cardiopulmonary resuscitation necessitated escalation to an ECMELLA strategy to re-establish circulation. ECLS was successfully weaned, and the device was explanted. After 31 days of Impella support, complications prompted the implantation of a HeartMate 3 (HM3) for long-term stabilization.

After a long course of rehabilitation with intensive physiotherapy and pulmonary weaning, the patient was discharged home 9 months after her initial surgery.

## Case 8

A 71-year-old woman experienced out-of-hospital cardiac arrest, prompting rapid initiation of extracorporeal cardiopulmonary resuscitation. Coronary angiography ruled out coronary obstruction.

MCS was established with ECLS and a femoral Impella CP. ECLS was successfully weaned after 4 days, and the Impella CP was removed after 7 days. After discontinuation of sedation, there was no sufficient awakening response. cCT imaging revealed subacute infarctions in the right frontal and cerebellar region. Over the course, the patient showed a sufficient awakening response and was later transferred to a neurological early rehabilitation facility.

Seven months later, after full rehabilitation, mitral valve reconstruction was performed. The echocardiography follow-up at 2 months showed a normal heart status.

## Case 9

A woman, aged 40, suffered cardiac arrest under anesthesia at the dentist. Return of spontaneous circulation was established after 5 minutes of cardiopulmonary resuscitation and 1 mg epinephrine. At the external hospital, TTE showed a severely impaired LV function. An Impella CP system was implanted via the right femoral artery. Due to high catecholamine demand, the patient was transferred to our clinic.

The patient underwent Impella 5.5 implantation via the right axillary artery. Additionally, ECLS support was established via the right jugular vein and right axillary artery (ECMELLA 2.1). Under ECMELLA 2.1 support, the patient’s hemodynamic condition improved, and catecholamine administration was reduced.

Subsequently, the patient underwent surgery for aortic root reconstruction, replacement of the ascending aorta, and stenting of the descending aorta due to type A aortic dissection. Additionally, the patient developed multiple organ failure with acute respiratory distress syndrome and severe coagulopathy. Implantation of a second venous cannula for ECLS support to improve drainage was performed. Despite these measures, sufficient blood flow (max 3 liters/min) could not be established with ECLS. We observed increasing lactate levels with insufficient ECLS flow. The patient expired 2 days after the initial presentation.

## Discussion

TTS accounts for 2% to 3% of patients presenting with ACS and is characterized by acute transient LV systolic dysfunction, which by definition is reversible.^19^ When TTS leads to CS, MCS can be utilized to bridge patients until cardiac recovery occurs. Since MCS has demonstrated favorable outcomes in the treatment of TTS and is able to facilitate LV unloading and provide circulatory support while decreasing the demand for catecholamines and inotropes, it has become an increasingly utilized strategy.^20^, ^21^

This large single–center retrospective study provides a detailed assessment of 9 patients from our clinic who were diagnosed with TTS and treated with MCS.

All patients underwent implantation of a microaxial LV assist device (Impella) due to its advantage of increasing coronary and end-organ perfusion while also effectively providing LV unloading. Our positive experience with the use of Impella and the cardiac recovery displayed by patients reflects the results reported by previous publications.^11^, ^12^, ^22^

Veno-arterial ECMO was not typically used as a sole MCS strategy for TTS, since LV afterload is increased due to retrograde perfusion, potentially further aggravating LV end-diastolic pressure.^20^, ^23^ When MCS provided by Impella was insufficient and full circulatory support was needed, combined Impella and ECMO support (ECMELLA concept) was established. Four patients in our study required escalation to an ECMELLA strategy to sufficiently improve cardiac output; of these, 2 did not survive. ECMELLA is not used to initiate MCS, since the use of multiple devices may increase the risk of complications.^23^

Owing to its multifaceted pathogenesis and potential triggers, TTS management remains difficult and can be further exacerbated by additional cardiovascular complications or complications occurring due to MCS therapy itself. In our study, 7 out of 9 patients experienced complications over the course of treatment. These complications include, but are not limited to, aortic dissection, hemorrhage, tachyarrhythmias, and hypoxic brain injury.

Though the optimal timing for weaning remains uncertain, the required duration of MCS is typically brief, given the transient nature of LV dysfunction. Because the risk of complications increases with longer support time, MCS duration should be limited to the shortest possible period.^10^, ^11^, ^20^

## Limitations

We would like to highlight several points that limit the results of this retrospective study. One main limiting factor is the retrospective and nonrandomized nature of this study.

We would like to note that there is no standardized treatment protocol for TTS-CS management, with treatment decisions being at the discretion of the treating physicians. Although all patients received care at our clinic, some patients were transferred from an external clinic after being diagnosed with TTS. As such, the availability of individual patient data concerning baseline vital and laboratory parameters was inconsistent. This is the case for baseline arterial lactate values provided in Tables 1 and 2, with measurements being taken at various time points after admission, sometimes after Impella implantation.

**Table 2:** Individual Patient Data Concerning Age, Diagnostic Workup, Symptoms, MCS Strategy, Ventricular Function, and Laboratory Parameters

Case	Age	Survival	Diagnosis	Symptoms	MCS strategy	LVEF baseline (%)	LVEF on MCS (%)	LVEF post MCS (%)	Lactate at admission (mg/dl)	LDH at admission (U/liter)	MCS duration (days)
1	67	Yes	CA, TTE	CS	Impella 2.5	25	35	NA	10^a^	1207	4
2	53	Yes	CA, TTE	Presyncope, vertigo	Impella 5.5	25	45	45	10^a^	189	4
3	77	Yes	CA, TTE	CS	Impella 5.5	33	NA	50	8^a^	370	5
4	57	Yes	CA, TTE	Dyspnea	Impella 5.5	NA	NA	63	8^a^	NA	8
5	76	No	CA, TTE	Palpitations, dyspnea	Impella	10	55	NA	43	NA	7
6	69	No	CA, TTE	CS	Ecmella	36	NA	NA	NA	NA	12
7	34	Yes	CA, TTE	CS	Ecmella 1.0 à HM3	17	19	Impaired	7^a^	418	ECMO 2 days, Impella 31 days, HM3 ongoing
8	71	Yes	CA, TEE	CS	Ecmella	70	70	60	81	NA	ECMO 4 days, Impella 7 days
9	40	No	CA, TTE	CS	Ecmella 2.1	10	No pump function	NA	99	541	2

Abbreviations: CA, coronary angiography; CS, cardiogenic shock; ECMO, extracorporeal membrane oxygenation; LDH, lactate dehydrogenase; LVEF, left ventricular ejection fraction; MCS, mechanical circulatory support; NA, not available; TTE, transthoracic echocardiography.

Data specifying the Impella system used in case 5 was not available.

a Lactate levels measured after Impella implantation.

## Conclusion

This retrospective study assessed a heterogeneous group of patients who showed typical imaging findings of TTS and required MCS due to severe CS. In this patient cohort, the use of microaxial LV assist devices (Impella) showed a reversal of the typical Takotsubo findings and an improvement in heart function. Patients who needed more blood flow required an escalation to an ECMELLA strategy to further improve cardiac output, while continuing LV unloading to protect the left ventricle from the increased afterload. Given the limited cohort size, further prospective studies are needed to provide better recommendations on the use of MCS (particularly Impella) for the treatment of TTS to increase survival and decrease catecholamine use.

## CRediT authorship contribution statement

**Anna Huang:** Methodology, Investigation, Data curation, Writing – original draft. **Johanna K. R. von Mackensen:** Methodology, Data curation, Writing – review & editing. **Vanessa I. T. Zwaans:** Writing – review & editing. **Carla L. Schuering:** Writing – review & editing. **Jasper Iske:** Writing – review & editing. **Leonard Pitts:** Writing – review & editing. **Julius Kaemmel:** Writing – review & editing. **Gaik Nersesian:** Writing – review & editing. **Cheng-Ying Chiu:** Writing – review & editing. **Christoph T. Starck:** Writing – review & editing. **Joerg Kempfert:** Writing – review & editing. **Stephan Jacobs:** Writing – review & editing. **Volkmar Falk:** Writing – review & editing. **Evgenij V. Potapov:** Writing – review & editing. **Leonhard Wert:** Conceptualization, Supervision, Writing – review & editing.

## Declaration of Competing Interest

**Christoph T. Starck:** Payment to his institution related to his activity in the form of speaker fees, honoraria, consultancy, advisory board fees, investigator, committee member of AngioDynamics, Abiomed, Medtronic, Spectranetics, Biotronik, LivaNova (Sorin), and Cook Medical and departmental or institutional research funding from Cook Medical. **Joerg Kempfert:** Grants or contracts from any entity: Edwards, LivaNova. Payment or honoraria for lectures, presentations, speakers bureaus, manuscript writing, or educational events: Edwards, Medtronic, Abbott, LivaNova, CryoLife. Leadership or fiduciary role in other board, society, committee, or advocacy group, paid or unpaid: TC EACTS, ECSC Board, ISMICS Board. **Volkmar Falk:** Grants or contracts from any entity: Medtronic GmbH, Biotronik SE & Co., Abbott GmbH & Co. KG, Boston Scientific, Edwards Lifesciences, Berlin Heart, Novartis Pharma GmbH, JOTEC/Cryolife GmbH, LivaNova, Zurich Heart. I hereby declare that I have relevant (institutional) financial activities outside the submitted work with the mentioned commercial entities in relation to educational grants (including travel support), fees for lectures and speeches, fees for professional consultation, research and study funds. **Evgenij V. Potapov:** Consulting fees: Abbott (institutional grants), Medtronic (institutional grants), Abiomed (institutional grants). Payment or honoraria for lectures, presentations, speakers bureaus, manuscript writing, or educational events: Abbott (institutional grants), Medtronic (institutional grants), Abiomed (institutional grants). Support for attending meetings and/or travel: Abbott (institutional grants), Medtronic (institutional grants), Abiomed (institutional grants). Participation in a Data Safety Monitoring Board or Advisory Board: Abbott, Medtronic. The other authors declare that they have no known competing financial interests or personal relationships that could have appeared to influence the work reported in this paper.

## Footnotes

Appendix A Supplementary data associated with this article can be found in the online version at doi:10.1016/j.jhlto.2026.100520.

## References

[1] Tranter M.H., Wright P.T., Sikkel M.B., Lyon A.R. (2013). Takotsubo cardiomyopathy: the pathophysiology. Heart Fail Clin.

[2] Lyon A.R., Bossone E., Schneider B. (2016). Current state of knowledge on Takotsubo syndrome: a Position Statement from the Taskforce on Takotsubo Syndrome of the Heart Failure Association of the European Society of Cardiology. Eur J Heart Fail.

[3] Stiermaier T., Eitel C., Desch S. (2016). Incidence, determinants and prognostic relevance of cardiogenic shock in patients with Takotsubo cardiomyopathy. Eur Heart J Acute Cardiovasc Care.

[4] Wittstein I.S., Thiemann D.R., Lima J.A. (2005). Neurohumoral features of myocardial stunning due to sudden emotional stress. N Engl J Med.

[5] Kato K., Lyon A.R., Ghadri J.-R., Templin C. (2017). Takotsubo syndrome: aetiology, presentation and treatment. Heart.

[6] Madhavan M., Rihal C.S., Lerman A., Prasad A. (2011). Acute heart failure in apical ballooning syndrome (TakoTsubo/stress cardiomyopathy): clinical correlates and Mayo Clinic risk score. J Am Coll Cardiol.

[7] Ghadri J.R., Cammann V.L., Jurisic S. (2017). A novel clinical score (InterTAK Diagnostic Score) to differentiate takotsubo syndrome from acute coronary syndrome: results from the International Takotsubo Registry. Eur J Heart Fail.

[8] Schneider B., Athanasiadis A., Schwab J. (2014). Complications in the clinical course of Tako-tsubo cardiomyopathy. Int J Cardiol.

[9] Vallabhajosyula S., Dunlay S.M., Murphree D.H. (2019). Cardiogenic shock in Takotsubo cardiomyopathy versus acute myocardial infarction: an 8-year national perspective on clinical characteristics, management, and outcomes. JACC: Heart Fail.

[10] Mariani S., Richter J., Pappalardo F. (2020). Mechanical circulatory support for Takotsubo syndrome: a systematic review and meta-analysis. Int J Cardiol.

[11] Napp L.C., Westenfeld R., Møller J.E. (2022). Impella mechanical circulatory support for Takotsubo syndrome with shock: a retrospective multicenter analysis. Cardiovasc Revasc Med.

[12] Napp L.C., Vogel-Claussen J., Schäfer A. (2017). First-in-man fully percutaneous complete bypass of heart and lung. JACC Cardiovasc Interv.

[13] Webster K.T., Apridonidze T., Mopala P.R. (2019). Stress-induced cardiomyopathy complicated by dynamic left ventricular outflow obstruction, cardiogenic shock, and ventricular septal rupture. Can J Cardiol.

[14] Grigoriadis C.E., Cork D.P., Dembitsky W., Jaski B.E. (2019). Recurrent cardiogenic shock associated with cannabis use: report of a case and review of the literature. J Emerg Med.

[15] von Mackensen J.K.R., Zwaans V.I.T., El Shazly A. (2024). Mechanical circulatory support strategies in Takotsubo syndrome with cardiogenic shock: a systematic review. J Clin Med.

[16] Elapavaluru S., Gologorsky A., Thai N. (2017). Perioperative stress cardiomyopathy in simultaneous liver and kidney transplantation: a call for early consideration of mechanical circulatory support. J Cardiothora Vasc Anesth.

[17] Bashline M.J., Rhinehart Z., Kola O., Fowler J., Kaczorowski D., Hickey G. (2022). Impella 5.0 is associated with a reduction in vasoactive support and improves hemodynamics in cardiogenic shock: a single-center experience. Int J Artif Organs.

[18] Ghadri J.R., Wittstein I.S., Prasad A. (2018). International expert consensus document on Takotsubo syndrome (part II): diagnostic workup, outcome, and management. Eur Heart J.

[19] Singh T., Khan H., Gamble D.T., Scally C., Newby D.E., Dawson D. (2022). Takotsubo syndrome: pathophysiology, emerging concepts, and clinical implications. Circulation.

[20] von Mackensen J.K.R., Shazly A.E., Schoenrath F. (2023). Successful treatment of cardiogenic shock due to Takotsubo syndrome with implantation of a temporary microaxial left ventricular assist device in transaxillary approach. J Cardiothorac Surg.

[21] Desai A., Ruiz J., Shapiro A., Klingbeil R., Martin A., Goswami R. (2025). Empowering early recovery: the role of Impella 5.5 in Takotsubo cardiomyopathy complicated by cardiogenic shock. J Clin Med.

[22] Nishikawa R., Nagano N., Kokubu N. (2021). Favorable effects of Impella on Takotsubo syndrome complicated with cardiogenic shock case series with successful resuscitation. Int Heart J.

[23] Schrage B., Becher P.M., Bernhardt A. (2020). Left ventricular unloading is associated with lower mortality in patients with cardiogenic shock treated with venoarterial extracorporeal membrane oxygenation: results from an international, multicenter cohort study. Circulation.

